# X-Linked Chronic Granulomatous Disease: Initial Presentation with Intracranial Hemorrhage from Vitamin K Deficiency in Infant

**DOI:** 10.1155/2018/7041204

**Published:** 2018-06-24

**Authors:** Boonchai Boonyawat, Yiwa Suksawat, Punchama Pacharn, Piradee Suwanpakdee, Chanchai Traivaree

**Affiliations:** ^1^Division of Genetics, Department of Pediatrics, Phramongkutklao Hospital and College of Medicine, Bangkok, Thailand; ^2^Division of Allergy and Immunology, Department of Pediatrics, Phramongkutklao Hospital and College of Medicine, Bangkok, Thailand; ^3^Division of Allergy and Immunology, Department of Pediatrics, Faculty of Medicine, Siriraj Hospital, Mahidol University, Bangkok, Thailand; ^4^Division of Neurology, Department of Pediatrics, Phramongkutklao Hospital and College of Medicine, Bangkok, Thailand; ^5^Division of Hematology and Oncology, Department of Pediatrics, Phramongkutklao Hospital and College of Medicine, Bangkok, Thailand

## Abstract

Vitamin K deficiency bleeding (VKDB) is a life-threatening condition and can be found in children as early as neonatal period with early onset intracranial hemorrhage (ICH). Here, we reported a 1-year-old boy who initially presented with intracranial hemorrhage secondary to vitamin K deficiency since 3 months of age and later found to have XL-CGD which was complicated by malabsorption due to severe vaccine-associated mycobacterial disease.

## 1. Introduction

Chronic granulomatous disease (CGD) is a rare genetically heterogenous primary immunodeficiency disease resulting from a defect of the nicotinamide adenine dinucleotide phosphate (NADPH) oxidase complex. The genetic defect leads to inability of phagocytes to generate superoxide and mediate intracellular microbe killing. The prevalence of CGD is approximately one in 200,000–250,000 live births worldwide without racial or ethnic predilection [1–3]. CGD is characterized by recurrent bacterial and fungal infections and chronic granuloma formation of the skin, lung, gastrointestinal tract, or lymph nodes and is caused by mutations in any one of five genes encoding essential subunits of the NADPH oxidase enzyme complex including gp91-*phox*, p22-*phox*, p47-*phox*, p67-*phox*, and p40-*phox*.

X-linked CGD (XL-CGD and OMIM 306400) is caused by mutations in the *CYBB* gene (OMIM 300481) which accounted for approximately 70% of CGD patients [2–4]. The *CYBB* gene comprises of 13 exons spanning 30 kb of genomic DNA and is located on Xp21.1. This gene encodes gp91-*phox* subunit, a key transmembrane protein in the NADPH oxidase complex. To date, over 700 different mutations in the *CYBB* gene have been reported in the HGMD database (http://www.hgmd.cf.ac.uk/ac/gene.php?gene:CYBB).

Here, we report a 1-year-old boy affected with XL-CGD who firstly presented with intracranial hemorrhage from vitamin K deficiency. Molecular analysis revealed a hemizygous c.676C > T (p.Arg226Ter) nonsense mutation in exon 7 of the *CYBB* gene which was inherited from his carrier mother.

## 2. Case Presentation

**Figure 1:**
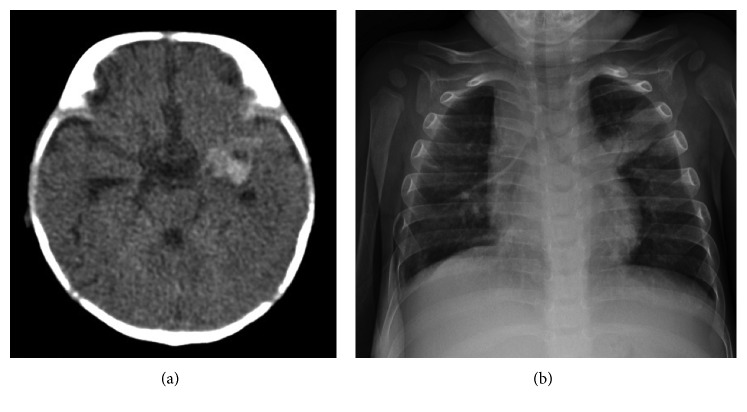
(a) CT brain showed hyperdensity lesion size of 1.5 × 1.8 cm at the left temporal lobe. (b) Chest X-ray revealed patchy infiltration at the left upper lobe.

A one-year-old Thai boy was referred to Phramongkutklao Hospital due to subacute fever, abdominal distension, mucous diarrhea, and failure to thrive. He was born at term with uneventful pregnancy, and he is the first child of nonconsanguineous parents. There was no history of autoimmune or primary immunodeficiency disorders in the family. Intradermal BCG vaccination was inoculated at the left buttock without any reaction within 3 months of life, and intramuscular vitamin K was routinely given after birth. He was exclusively breastfed. At 3 months of age, he developed frequent vomiting and irritability. Physical examination revealed enlarged and tense anterior fontanelle. CT brain showed hyperdensity lesion size of 1.5 × 1.8 cm at left temporal lobe with perilesional edema (Figure 1(a)) which was confirmed to be intracerebral hemorrhage. All hematologic, coagulation studies and biochemical laboratory tests (Table 1) were consistent with deficiency of vitamin K dependent clotting factors. The cause of vitamin K deficiency in this patient was presumed to be caused by malabsorption mechanism. Therefore, intravenous vitamin K was given for 3 days at initial presentation, and the coagulogram data was corrected within 24 hours. One week later, the patient developed steatorrhea. Fat malabsorption was suspected as the levels of fat-soluble vitamins were evaluated (Table 1). Cystic fibrosis was excluded by the negative sweat chloride test. At 4 months of age, perianal abscess was detected and treated with amoxicillin/clavulanic acid for 7 days without surgical drainage. However, subsequent pus culture was not performed. At the age of 6 months, lymphadenopathy of 3 cm in size at the left groin was detected. Fine needle aspiration was accordingly performed, and pus culture was found to be positive for BCG and the tuberculin skin test was positive at 15 × 20 mm. Chest X-ray revealed no pulmonary infiltration. The patient was diagnosed with BCG lymphadenitis and was treated with isoniazid and rifampicin. Interestingly, there was no history of tuberculosis contact in the family. Nevertheless, the patient did lose to follow-up which resulted in the delay of definite diagnosis in this patient.

**Figure 2:**
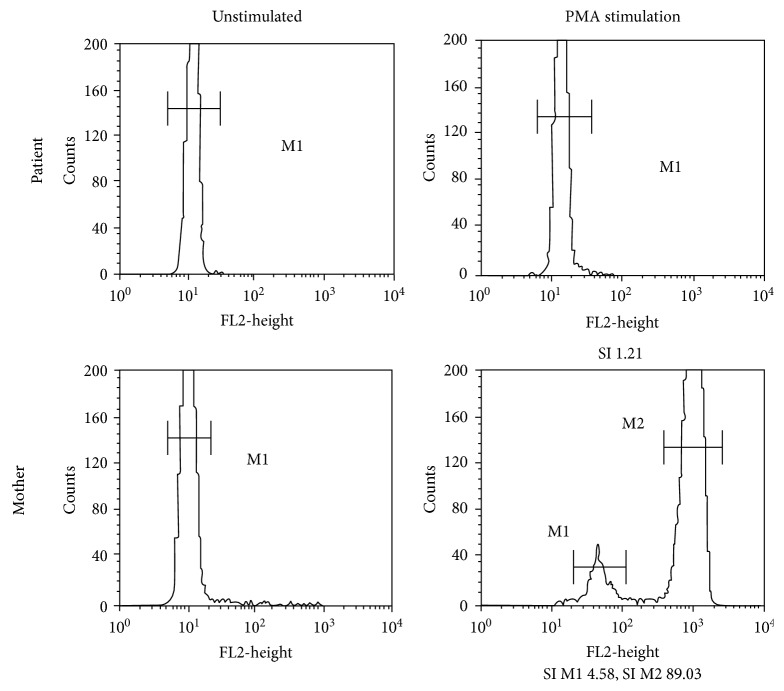
The DHR assay of the patient showed complete absence of DHR shift after granulocyte stimulation. The SI was 1.21 which was compatible with XL-CGD. The DHR assay of the patient's mother showed bimodal distribution which was compatible with the XL-CGD carrier. The stimulation index (SI) was calculated by dividing the mean channel of fluorescence intensity in the stimulated cell over the mean channel of fluorescence intensity in the unstimulated cell.

On physical examination at age of 1 year, his weight was 7.8 kg (<3rd percentile) and height was 69.5 cm (<3rd percentile). Abdominal distension, moderate hepatosplenomegaly, and ascites were detected. Left inguinal lymph node was still palpated with 1.5 cm in size. The site of BCG vaccination showed no induration. Physical examinations were unremarkable. Hematologic and biochemical laboratory tests were described (Table 1), and chest radiography showed consolidation at the left upper lobe (Figure 1(b)). Abdominal CT showed generalized ascites with evidence of hepatosplenomegaly. Abdominal paracentesis was performed, and the results were described (Table 1). Ascitic fluid adenosine deaminase (ADA) was performed because of suspicious of mycobacterial infection, and the result was compatibly high. The ascitic fluid PCR was positive for BCG. Disseminated BCG infection was diagnosed in our patient. IgG was slightly elevated (1,236 mg/dL) while IgM and IgA levels were normal (Table 1). Lymphocyte subset analyses revealed normal T-cell and B-cell counts. The neutrophil dihydrorhodamine (DHR) test revealed no fluorescence detection after granulocyte stimulation. The stimulation index (SI) was 1.21 which was compatible with XL-CGD (Figure 2).

Finally, the patient was diagnosed with XL-CGD accompanied with disseminated BCG infection. This clarified the clinical of fat malabsorption leading to vitamin K deficiency since 3 months of age. Treatment was started with antituberculosis including isoniazid, rifampin, pyrazinamide, ethambutol, and amikacin. Itraconazole and co-trimoxazole were given as the prophylactic treatment. The clinical of ascites and steatorrhea was improved after 2 weeks of treatment. The DHR assay was also performed on the mother and revealed bimodal distribution compatible with the XL-CGD carrier (Figure 2). Allogeneic hematopoietic stem cell transplantation (HCT) is therefore planned as the curative treatment since the mother is six months pregnant.

### 2.1. Mutation Analysis of the *CYBB* Gene

After informed consent was obtained from the patient's parents, genomic DNA was extracted from peripheral blood leukocytes using the commercial available kit as per the manufacturer's protocol. Thirteen coding exons and exon-intron boundaries of the *CYBB* gene were amplified by PCR using specific of primers for each exon as previously described [5]. The PCR products were purified and directly sequenced in both forward and reverse directions. The reference sequences were NM_00397.3 for *CYBB* cDNA and NP_000388.2 for gp19-*phox* protein.

## 3. Results

**Figure 3:**
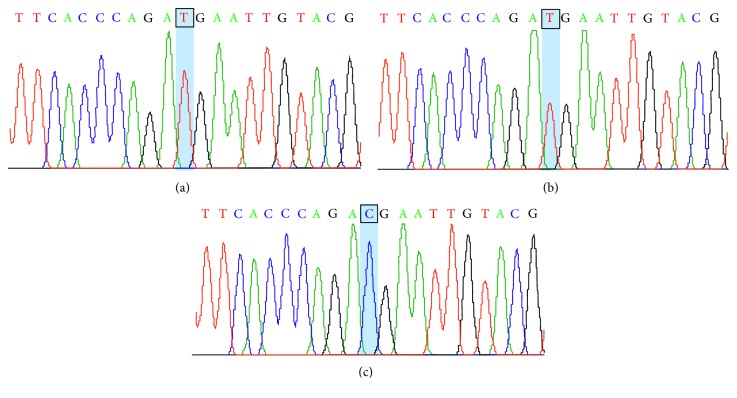
Electropherogram of the exon 7 of *CYBB* gene revealed a hemizygous c.676C > T (p.Arg226Ter) nonsense mutation in the patient's DNA (a), a heterozygous for the same mutation in the maternal DNA (b), and normal DNA sequence (c).

Mutation analysis by direct DNA sequencing of all 13 coding exons and exon-intron junctions of the *CYBB* gene revealed a hemizygous; c.676C > T or p.Arg226Ter, nonsense mutation which is located in exon 7 of the *CYBB* gene (Figure 3). The maternal DNA was identified to carry a heterozygous of the same mutation suggesting that this mutation was inherited from the carrier mother.

## 4. Discussion

Chronic granulomatous disease (CGD) is a primary immunodeficiency disorder involving defective phagocytic function as a result of absence or reduced NADPH oxidase complex. X-Linked Chronic Granulomatous Disease formed (XL-CGD) is the most common and account for approximately 70% of the patients [2–4]. XL-CGD is caused by mutations in the *CYBB* gene encoding gp91-*phox* protein. Almost all CGD patients have recurrent bacterial and fungal infections and chronic granuloma formation. In this study, we reported a 1-year-old boy suffering from XL-CGD who firstly presented at 3 months of age with intracranial hemorrhage from vitamin K deficiency without any evidence of infections. When combination with steatorrhea and low level of other fat-soluble vitamins, these indicated fat malabsorption in our patient.

Gastrointestinal (GI) involvement occurs in approximately 50% of patients with CGD. It usually affects patients with x-linked inheritance more than the autosomal recessive [5]. Most GI problems are results from granulomatous colitis, followed by protein-losing enteropathy and inflammatory disease [1, 5, 6]. Gastrointestinal malabsorption in this case is probably caused by intestinal BCG infection which was supported by the development of inguinal BCG lymphadenitis at the age of 6 months. Normally, BCG vaccine was routinely given on the left shoulder in Thai infants, but it was injected on the left buttock in our patient at a private hospital because his mother preferred for cosmetic reason. This explained why our patient had the regional BCG lymphadenitis at left inguinal node and the distant intestinal BCG infection. Primary immunodeficiency (PID) was suspected at the age of 1 year according to disseminated BCG infection involving liver, spleen, and lungs. XL-CGD was diagnosed by DHR flow cytometry assay which is a rapid and sensitive screening test for CGD [7]. Most XL-CGD patients demonstrate no activity of DHR assay as in our patient. From the largest cohort study of XL-CGD in China, majority of the patients (90%) had the symptom debut at age 3 months of life and about one-half of patients were diagnosed after 1 year of age [4]. The most prevalent infection sites were lungs, gastrointestinal tract, and lymph nodes as also shown in our patient. Perianal abscess which is the most frequent gastrointestinal manifestation in XL-CGD was also presented in our patient [4].

Bacille Calmette-Guérin (BCG) vaccinations were routinely given to all Thai neonates at birth as part of the national vaccination program recommended by the World Health Organization (WHO), since Thailand is a country with high prevalence of tuberculosis. BCG vaccination is harmless in most children; however, BCG infection has been occasionally reported with the incidence of 1 : 10,000–100,000 [8]. Primary immunodeficiency (PID) is the major risk factor of BCG infection. Approximately 50–75% of BCG infection patients had definite PID [9, 10]. While CGD is the most common (75%) type of PID causing BCG infection in Chinese children. Severe combined immunodeficiency (SCID) is the most common PID in children with BCGitis or disseminated BCG infection in Caucasians [9, 10]. XL-CGD makes our patient susceptible to BCG infection at the earlier onset age than in normal individuals, since respiratory burst of phagocytic cells plays an important role in host defense mechanism against mycobacterium infections [9, 10].

The *CYBB* gene contains 13 exons and encodes 570-aa gp91-*phox* protein. The gp91-*phox* consists of 4 domains including N-terminal domain (aa 1–277), FAD-binding domain (aa 278–397), NADPH binding domain (aa 398–483 and 504–570), and a loop over NADPH binding domain (aa 484–503). To date, over 700 *CYBB* mutations have been identified throughout 13 exons and exon-intron boundaries, and majority of the mutations was unique [4, 11]. Most common mutations leading to XL-CGD is single nucleotide substitutions including missense, nonsense, and splice-site mutations, followed by deletions, insertions, and combination of small deletions and insertions. Approximately 60% of the mutation is located in the N-terminal domain. Mutation analysis of the *CYBB* gene was performed to confirm the diagnosis of XL-CGD in our patient and identified a hemizygous for c.676C > T (p.Arg226Ter) mutation in exon 7 of the gene. This nonsense mutation is located in the N-terminal domain of gp91-*phox*, resulting in a total absence of NADPH oxidase activity in activated neutrophils and is associated with a severe phenotype as in our patient. Although this mutation is reported in Thai patient for the first time, it has been described in many XL-CGD patients from various countries in Asia, Europe, and America suggesting the hot spot of this mutation [3, 4, 9, 11, 12]. DHR assay and molecular analysis also confirmed that the mutation identified in our patient was inherited from his carrier mother. Only one third of individuals with XL-CGD occurred de novo [4, 11]. In Thailand, there were 2 previous reports of molecular confirmed XL-CGD patients [13, 14]. All patients presented with recurrent bacterial and fungal infections with abscess formation in lungs, liver, spleen, lymph nodes, and skin. None of them presented with other BCGitis or disseminated BCG infection and intracranial hemorrhage. The prophylactic use of IFN-gamma remains variable. The previous study [15] showed that long-term prophylaxis with IFN-gamma did not significantly change the rate of total infection per patient-year compared to the control, and the group determined that there was no evidence to justify long-term prophylaxis with IFN-gamma.

In our country, the IFN-gamma is not available. Allogeneic HCT is the only curative treatment available for CGD and may reverse both infectious and inflammatory complications. Importantly, several studies have demonstrated that outcome with 10/10 matched unrelated donor (MUD) is comparable to those with match sibling donors (MSD). It was proposed that more myelaoblative conditioning (MAC) may be necessary for stable engraftment. Given the rarity of the disease, the conditioning regimen reported in the literatures was varied with the average overall survival from 83–100% and disease-free survival from 33–89% [16–18].

In conclusion, we reported an XL-CGD patient who firstly presented with intracranial hemorrhage from vitamin K deficiency in an infant which was the result of fat malabsorption. BCGitis, and dissiminated BCG infection was subsequently diagnosed and possibly was the cause of this malabsorption. Molecular information is also helpful for proper genetic counseling including prenatal diagnosis and preimplantation genetic diagnosis to the patient and the family.

## Figures and Tables

**Table 1:** Laboratory parameters.

Onset	Laboratory findings
*At 3-month old*	
Complete blood count (CBC)	Hb 8.2 g/dl, WBC 17,700/mm^3^ (P 35%, L 60%, monocyte 5%), platelets 521,000/mm^3^
Coagulation studies	PT 63.8 sec (*N* 11–14), INR 5.23 aPTT 69.5 sec (*N* 23–31), TT 10.6 sec (*N* 8.7–11.1)
Factor assay (% activity)	FII 5.50% (*N* 70–140), FVII 10.90% (*N* 70–140) FIX 7.10% (*N* 50–200), FX 4.70% (*N* 50–200) FV 121% (*N* 70–130), FVIII 148.1% (*N* 50–200)
Liver function test	AST 55 U/L, ALT 15 U/L, albumin 4.2 g/dL, total birilubin 0.6 mg/dL, direct birilubin 0.3 mg/dL
Fat-soluble vitamins level	Vitamin A 0.87 *µ*mol/L (*N* 0.8–2.9), vitamin E 14.70 *µ*mol/L (*N* 8–43), vitamin D (25-OH) 7.51 ng/mL (*N* 30–100)
*At 1-year old*	
Complete blood count (CBC)	Hb 7.8 g/dl, WBC 16,200/mm^3^ (P 92%, L 6%, monocyte 2%), platelets 427,000/mm^3^
Coagulation studies	PT 11 sec (*N* 11–14), INR 1.1 aPTT 26 sec (*N* 23–31), TT 10 sec (*N* 8.7–11.1)
Liver function test	AST 45 U/L, ALT 17 U/L, albumin 2.5 g/dL, total birilubin 0.6 mg/dL, direct birilubin 0.3 mg/dL
Immunological studies	IgG 1,236 mg/dL (*N* 700–1,000), IgM 161 mg/dL (*N* 40–230), IgA 102 mg/dL (*N* 70–400)
Ascitic fluid parameter	Straw color, serum ascites albumin gradient (SAAG) 0.5 g/dL, WBC 1,600/mm^3^ with 100% mononuclear cell adenosine deaminase (ADA) 83 U/L (*N* 0–33)

## References

[1] Winkelstein J. A., Marino M. C., Johnston R. B. (2000). Chronic granulomatous disease: report on a national registry of 368 patients. *Medicine*.

[2] van den Berg J. M., van Koppen E., Ahlin A. (2009). Chronic granulomatous disease: the European experience. *PLoS One*.

[3] Ishibashi F., Nunoi H., Endo F., Matsuda I., Kanegasaki S. (2000). Statistical and mutational analysis of chronic granulomatous disease in Japan with special reference to gp91-phox and p22-phox deficiency. *Human Genetics*.

[4] Wu J., Wang W. F., Zhang Y. D., Chen T. X. (2017). Clinical Features and genetic analysis of 48 patients with chronic granulomatous disease in a Single Center Study from Shanghai, China (2005–2015): new studies and a literature review. *Journal of Immunology Research*.

[5] Marciano B. E., Rosenzweig S. D., Kleiner D. E. (2004). Gastrointestinal involvement in chronic granulomatous disease. *Pediatrics*.

[6] Huang A., Abbasakoor F., Vaizey C. J. (2006). Gastrointestinal manifestations of chronic granulomatous disease. *Colorectal Disease*.

[7] Jirapongsananuruk O., Malech H. L., Kuhns D. B. (2003). Diagnostic paradigm for evaluation of male patients with chronic granulomatous disease, based on the dihydrorhodamine 123 assay. *Journal of Allergy and Clinical Immunology*.

[8] Grange J. M. (1998). Complications of bacille Calmette-Guerin (BCG) vaccination and immunotherapy and their management. *Communicable Disease and Public Health*.

[9] Ying W., Sun J., Liu D. (2014). Clinical characteristics and immunogenetics of BCGosis/BCGitis in Chinese children: a 6 year follow-up study. *PLoS One*.

[10] Norouzi S., Aghamohammadi A., Mamishi S., Rosenzweig S. D., Rezaei N. (2012). Bacillus Calmette-Guerin (BCG) complications associated with primary immunodeficiency diseases. *Journal of Infection*.

[11] Roos D., Kuhns D. B., Maddalena A. (2010). Hematologically important mutations: X-linked chronic granulomatous disease (third update). *Blood Cells, Molecules and Diseases*.

[12] Jakobsen M. A., Katzenstein T. L., Valerius N. H. (2012). Genetical analysis of all Danish patients diagnosed with chronic granulomatous disease. *Scandinavian Journal of Immunology*.

[13] Vilaiphan P., Chatchatee P., Ngamphaiboon J., Tongkobpetch S., Suphapeetiporn K., Shotelersuk V. (2007). Nonsense mutations of the CYBB gene in two Thai families with X-linked chronic granulomatous disease. *Asian Pacific Journal of Allergy and Immunology*.

[14] Jirapongsananuruk O., Noack D., Boonchoo S. (2007). A novel mutation of the CYBB gene resulting in severe form of X-linked chronic granulomatous disease. *Asian Pacific Journal of Allergy and Immunology*.

[15] Martire B., Rondelli R., Soresina A. (2008). Clinical features, long-term follow-up and outcome of a large cohort of patients with chronic granulomatous disease: an Italian multicenter study. *Clinical Immunology*.

[16] Khandelwal P., Bleesing J. J., Davies S. M., Marsh R. A. (2016). A single-center experience comparing alemtuzumab, fludarabine, and melphalan reduced-intensity, conditioning with myeloablative busulfan, cyclophosphamide, and antithymocyte globulin for chronic granulomatous disease. *Biology of Blood and Marrow Transplantation*.

[17] Morillo-Gutierrez B., Beier R., Rao K. (2016). Treosulfan-based conditioning for allogeneic HSCT in children with chronic granulomatous disease: a multicenter experience. *Blood*.

[18] Gungor T., Teira P., Slatter M. (2014). Reduced-intensity conditioning and HLA-matched haemopoietic stem-cell transplantation in patients with chronic granulomatous disease: a prospective multicentre study. *The Lancet*.

